# Progress Toward Global Eradication of Dracunculiasis — Worldwide, January 2021–June 2022

**DOI:** 10.15585/mmwr.mm7147a2

**Published:** 2022-11-25

**Authors:** Donald R. Hopkins, Adam J. Weiss, Sarah Yerian, Sarah G.H. Sapp, Vitaliano A. Cama

**Affiliations:** ^1^The Carter Center, Atlanta, Georgia; ^2^World Health Organization Collaborating Center for Dracunculiasis Eradication, Division of Parasitic Diseases and Malaria, Center for Global Health, CDC.

Dracunculiasis (Guinea worm disease), caused by the parasite *Dracunculus medinensis*, is acquired by drinking water containing small crustacean copepods (water fleas) infected with *D. medinensis* larvae. Recent evidence suggests that the parasite also appears to be transmitted by eating fish or other aquatic animals. About 1 year after infection, the worm typically emerges through the skin on a lower limb of the host, causing pain and disability (*1*). No vaccine or medicine is available to prevent or treat dracunculiasis. Eradication relies on case containment* to prevent water contamination and other interventions to prevent infection, including health education, water filtration, treatment of unsafe water with temephos (an organophosphate larvicide), and provision of safe drinking water (*1*,*2*). CDC began worldwide eradication efforts in October 1980, and in 1984 was designated by the World Health Organization (WHO) as the technical monitor of the Dracunculiasis Eradication Program (*1*). In 1986, with an estimated 3.5 million cases^†^ occurring annually in 20 African and Asian countries^§^ (*3*), the World Health Assembly called for dracunculiasis elimination. The Guinea Worm Eradication Program (GWEP),^¶^ led by The Carter Center and supported by partners that include WHO, UNICEF, and CDC, began assisting ministries of health in countries with endemic disease. In 2021, a total of 15 human cases were identified and three were identified during January–June 2022. As of November 2022, dracunculiasis remained endemic in five countries (Angola, Chad, Ethiopia, Mali, and South Sudan); cases reported in Cameroon were likely imported from Chad. Eradication efforts in these countries are challenged by infection in animals, the COVID-19 pandemic, civil unrest, and insecurity. Animal infections, mostly in domestic dogs, some domestic cats, and in Ethiopia, a few baboons, have now surpassed human cases, with 863 reported animal infections in 2021 and 296 during January–June 2022. During the COVID-19 pandemic all national GWEPs remained fully operational, implementing precautions to ensure safety of program staff members and community members. In addition, the progress toward eradication and effectiveness of interventions were reviewed at the 2021 and 2022 annual meetings of GWEP program managers, and the 2021 meeting of WHO’s International Commission for the Certification of Dracunculiasis Eradication. With only 15 human cases identified in 2021 and three during January–June 2022, program efforts appear to be closer to reaching the goal of eradication. However, dog infections and impeded access because of civil unrest and insecurity in Mali and South Sudan continue to be the greatest challenges for the program. This report describes progress during January 2021–June 2022 and updates previous reports (*2*,*4*). 

In affected countries, the national GWEP receives monthly case reports from supervised volunteers in each village under active surveillance.** Villages in which endemic transmission has ended (i.e., zero human cases or animal infections reported for ≥12 consecutive months) are kept under active surveillance for 2 additional years. WHO certifies a country as dracunculiasis-free after adequate nationwide surveillance for ≥3 consecutive years with no indigenous human cases or animal infections.^††^

Since 1986, WHO has certified 199 countries, areas, and territories as dracunculiasis-free; only the five countries with endemic dracunculiasis, plus the Democratic Republic of the Congo and Sudan still lack certification.^§§^ Representatives of seven countries with current or former endemic dracunculiasis, the United Arab Emirates, The Carter Center, and WHO renewed their commitment to completing Guinea worm eradication at a Guinea Worm Summit in Abu Dhabi in March 2022.

In 2021, Chad, Ethiopia, Mali, and South Sudan reported 15 human cases compared with 27 human cases in 2020, the smallest number of human cases ever reported annually (Table 1). Cameroon, Chad, Ethiopia, and Mali reported 863 infected animals (mostly dogs), compared with 1,601 animal infections reported in 2020. During January–June 2022, three human cases and 296 infected animals were reported, representing 40% and 34% reductions in human cases and animal infections, respectively, compared with five human cases and 450 infected animals reported during January–June 2021 (Table 2). During January–June 2022, CDC received 20 specimens from humans for morphologic or molecular identification, including three (15%) that were laboratory-confirmed *D. medinensis*^¶¶^ (Table 3), compared with 13 specimens received and five (38%) confirmed during January–June 2021. During the first 6 months of 2022, CDC received 12 animal specimens, 10 of which were laboratory-confirmed *D. medinensis*, compared with six confirmed among 36 specimens received during January–June 2021. Previous genetic studies confirmed that Guinea worms collected from both animals and humans are *D. medinensis* (*5*). This activity was reviewed by CDC and was conducted consistent with applicable federal law and CDC policy.***

**TABLE 1:** Reported dracunculiasis human cases and animal infections, surveillance, and status of local interventions in villages with endemic disease, by country — worldwide, 2021

Human cases/Surveillance/Intervention status	Country					
	Chad*	Ethiopia	Mali^†^	South Sudan	Angola	Total
**Reported human cases**						
No. indigenous	8	1	2	4	0	**15**
No. imported^§^	0	0	0	0	0	**0**
% Contained^¶^ (no./total no.)	75 (6/8)	100	50 (1/2)	25 (1/4)	NA	**60 (9/15)**
% Change (no. change) in indigenous human cases in villages/localities under surveillance , 2020–2021	−38 (13 to 8)	−91 (11 to 1)	100 (1 to 2)	300 (1 to 4)	−100 (1 to 0)	**−44 (27 to 15)**
**Reported animal cases**						
No. indigenous	843**	3	17	0	0	**863**
No. imported^††^	0	0	0	0	0	**0**
% Contained^¶^ (no./total no.)	81 (681/843)	67 (2/3)	59 (10/17)	NA	NA	**80 (693/863)**
% Change (no. of cases in 2020) in indigenous animal infections in villages or localities under surveillance	−47 (1,577)	−80 (15)	89 (9)	NA	NA	**−46 (1,601)**
**Villages under active surveillance, 2021**						
No. of villages	2,358	198	2,216	2,174	61	**7,007**
No. (%) reporting monthly	2,358 (100)	198 (100)	2,216 (100)	1,975 (91)	8 (13)	**6,755 (96)**
No. reporting ≥1 human case	8	1	1	4	0	**14**
No. reporting only imported^††^ human cases	0	0	0	0	0	**0**
No. reporting indigenous human cases	8	1	1	4	0	**14**
No. reporting ≥1 animal infection	321	3	10	0	0	**334**
No. reporting only imported^††^ animal infections	0	0	0	0	0	**0**
No. reporting indigenous animal infections	321	3	10	0	0	**334**
**Status of interventions in villages with human dracunculiasis, 2021**						
No. of villages with human dracunculiasis, 2021	8	1	1	4	NA	**14**
% Reporting monthly^§§^ (no./total no.)	75 (6/8)	100 (1/1)	100 (1/1)	100 (4/4)	NA	**86 (12/14)**
% Filters in all households^§§^ (no./total no.)	38 (3/8)	100 (1/1)	100 (1/1)	100 (4/4)	NA	**64 (9/14)**
% Using temephos^§§^	75 (6/8)	100 (1/1)	100 (1/1)	100 (4/4)	NA	**86 (12/14)**
% With ≥1 source of safe water^§§^ (no./total no.)	38 (3/8)	100 (1/1)	100 (1/1)	25 (1/4)	NA	**43 (6/14)**
% Provided health education^§§^ (no./total no.)	75 (6/8)	100 (1/1)	100 (1/1)	100 (4/4)	NA	**86 (12/14)**
**Status of interventions in villages with animal dracunculiasis, 2021**						
No.of villages with animal dracunculiasis 2021	340	3	10	0	0	**353**
% Reporting monthly^§§^ (no./total no.)	95 (323/340)	100 (3/3)	100 (10/10)	NA	NA	**95 (336/353)**
% Using temephos^§§^ (no./total no.)	61 (208/340)	100 (3/3)	100 (10/10)	NA	NA	**63 (221/353)**
% Provided health education^§§^ (no./total no.)	95 (323/340)	100 (3/3)	100 (10/10)	NA	NA	**95 (336/353)**

**Abbreviations**: GWEP = Guinea Worm Eradication Program; NA = not applicable.

* Participants at the annual Chad GWEP review meeting in November 2014 adopted “1+ case village” as a new description for villages in Chad affected by human cases of Guinea worm disease or dogs infected with Guinea worms and defined it as “a village with one or more indigenous and imported cases of Guinea worm infections in humans, dogs, and cats in the current calendar year or previous year.”

^†^ Civil unrest and insecurity since a coup d'état in 2012 continued to constrain GWEP operations (i.e., supervision, surveillance, and interventions) in regions with endemic dracunculiasis (Gao, Kidal, Mopti, Segou, and Timbuktu) during January 2020 – June 2021.

^§^ Imported from another country.

^¶^ Human cases are contained when all of the following criteria are met: 1) infected patients are identified ≤24 hours after worm emergence; 2) patients have not entered any water source since worm emergence; 3) a village volunteer/health care provider properly treats the lesion until all detectable worms are fully removed and educates the patient not to contaminate water sources; 4) the containment process is validated by a GWEP supervisor ≤7 days after worm emergence; and 5) all contaminated and potentially contaminated sources of drinking water are treated with temephos.

** Chad reported 833 animal cases in 2021. Ten animal cases were reported from Cameroon in 2021, all in villages along the Chad-Cameroon border; these are believed to have been acquired in Chad.

^††^ Imported from another in-country disease-endemic village.

^§§^ The denominator is number of villages with endemic human dracunculiasis during 2020–2021.

**TABLE 2:** Number of reported indigenous dracunculiasis cases, by country –– worldwide, January 2020–June 2022

Country	No. (% contained)		% Change Jan–Dec 2020 to Jan–Dec 2021	No. (% contained)		% Change Jan–Jun 2021 to Jan–Jun 2022
	Jan–Dec 2020	Jan–Dec 2021		Jan–Jun 2021	Jan–Jun 2022	
**Human cases**						
Chad	12 (42)	8 (75)	−33	4 (75)	3 (33)	−25
Ethiopia	11 (100)	1 (100)	−91	1 (100)	0 (—)	−100
Mali*	1 (0)	2 (50)	100	0 (—)	0 (—)	NA
South Sudan	1 (100)	4 (25)	300	0 (—)	0 (—)	NA
Angola	1 (0)	0 (—)	−100	0 (—)	0 (—)	NA
Cameroon**^†^**	1 (100)	0 (—)	−100	0 (—)	0 (—)	NA
**Total**	**27 (67)**	**15 (60)**	−**44**	**5 (80)**	**3 (33)**	−**40**
**Animal infections** ^§^						
Chad	1,571 (81)	833 (81)	−47	438 (79)	264 (71)	−40
Ethiopia	15 (73)	3 (67)	−80	0 (—)	0 (—)	NA
Mali	9 (56)	17 (59)	89	2 (100)	2 (100)	0
Cameroon**^†^**	6 (100)	10 (100)	67	10 (100)	26 (100)	160
South Sudan	0 (—)	0 (—)	NA	0 (—)	0 (—)	NA
Angola	0 (—)	0 (—)	NA	0 (—)	7 (0)	NA
**Total**	**1,601 (81)**	**863 (80)**	−**46**	**450 (80)**	**299 (72)**	−**34**

**Abbreviations:** GWEP = Guinea Worm Eradication Program; NA = not applicable.

* Civil unrest and insecurity since a coup d'état in 2012 continued to constrain GWEP operations (i.e., supervision, surveillance, and interventions) in regions with endemic dracunculiasis (Gao, Kidal, Mopti, Segou, and Timbuktu) during January 2020–June 2021.

**^†^** One human case, 38 infected dogs, and one cat were detected in an area of Cameroon near the border with Chad during January 2020–June 2022; these infections are believed to have been acquired in Chad.

^§^ In 2021, Chad reported infections in dogs and some cats; Ethiopia reported infections in dogs, cats, and baboons; and Mali reported infections in dogs and one cat; in 2020, Angola reported infections in dogs and Cameroon in dogs and one cat.

**TABLE 3:** Characteristics of specimens from humans and animals received at CDC for laboratory diagnosis of *Dracunculus medinensis* — January 2021–June 2022

Characteristic	2021			2022
	Jan–Dec 2021	Jan–Jun 2021	Jul–Dec 2021	Jan–Jun 2022
**Human specimens**				
**Total no. received**	**45**	**13**	**32**	**20**
No. (%) positive*	14^†^ (31)	5 (38)	9^†^ (28)	3^†^ (15)
**Country of origin, no. of positive specimens (no. of patients)**				
Chad	7^†^ (7)	4 (4)	3 (3)	3 (3)
Ethiopia	1 (1)	1 (1)	—**^§^**	—
Mali	2 (2)	—	2 (2)	—
South Sudan	4 (4)	—	4 (4)	—
No (%) negative*	31 (68)	8 (62)	23 (72)	17 (85)
**Negative specimens, no. (%) of other laboratory identifications**				
*Onchocerca* sp.	—	—	—	6 (35)
Free-living nematode**^¶^**	8 (26)	2 (25)	6 (26)	1 (6)
Other parasitic nematode**	2 (6)	—	2 (9)	1 (6)
Sparganum	5 (16)	3 (38)	2 (9)	3 (18)
Cestode (non-sparganum)	1 (3)	—	1 (4)	—
Annelid	2 (6)	—	2 (9)	—
Animal-origin tissue	9 (29)	2 (25)	7 (30)	4 (24)
Plant material	2 (6)	—	2 (4)	2 (12)
Unknown origin	2 (6)	1 (13)	1 (4)	—
**Animal specimens**				
**Total no. received**	**73**	**36**	**37**	**12**
No. (%) positive*	38 (52)	6 (17)	32 (86)	10 (83)
**Positive specimens, country/species of origin, no. of specimens (no. of animals)**				
**Angola**				
Dog	—	—	—	6 (6)
**Cameroon**				
Dog	10 (10)	—	10	—
**Central African Republic**				
Dog	1 (1)	1	—	—
**Chad**				
Cat	2 (2)	—	2	2 (2)
Dog	4 (4)	2	2	—
Other animals (wildcats)	2 (2)	1	1	—
**Ethiopia**				
Cat	1 (1)	—	1	—
Dog	2 (2)	—	2	—
**Mali**				
Cat	1 (1)	—	1	—
Dog	15 (15)	2	13	2 (2)
No. (%) negative specimens*^,††^	35 (48) **	30 (83)	5 (14)	2 (17)^§§^

* Positive specimens were confirmed as *D. medinensis*; negative specimens were ruled out as *D. medinensis*.

^†^ CDC received 15 specimens from human cases in 2022 and 14 in 2021. The remaining specimen received in February 2022 was from a human case in Chad.

^§^ Dashes indicate no specimens received.

^¶^ Free-living nematodes primarily included adult Mermithidae and other nematodes identified as belonging to nonparasitic taxa. Other parasitic nematodes included non-*Onchocerca* nematodes identified as belonging to parasitic taxa.

** Other parasitic nematodes submitted in association with human cases during January–December 2021 included one ascarid, a *Eustrongylides-*like nematode; in 2022, the one other parasitic nematode was ascarid.

^††^ The 35 negative specimens from animals were identified as follows: 24 Filaroidea from which three were *Setaria* sp*.,* 12 were in the subfamily Dirofilariinae (e.g., *Dirofilaria, Skjrabinodera*), and nine were only identified at the superfamily level (Filarioidea); among the 11 remaining negative specimens five were free-living nematodes, two *Mastophorus* or *Protospirura-*like nematodes, one *Eustrongylides* sp., two were not identified past superfamily Spiruroidea, and one was a horsehair worm (Nematomorpha).

^§§^ One specimen was identified only at the superfamily level (Filarioidea), and the other was a nematode in the family Diplotriaenidae.

## Country Reports

**Angola.** Details of the unexpected discovery of dracunculiasis in three persons during 2018–2020 who had no history of foreign travel and in one dog in 2019 have been described. During March 29, 2020–March 28, 2022, no cases among humans or dogs were detected during ongoing active surveillance in 61 villages and routine integrated case searches (e.g., during National Immunization Days); seven infected dogs were detected during March–May 2022 in the same province as the previous human cases and dog infections (Table 2). Angola offers cash reward equivalent to US$450 for reporting an infected human or animal. Genetic analysis of Angola’s Guinea worm specimens did not help elucidate a source because no clear link to other previously analyzed *D. medinensis* was detected (*6*).

**Chad.** Chad reported eight human cases among eight villages in 2021 (Table 1). During the first half of 2022, Chad reported three human cases, compared with four human cases reported during January–June 2021. In 2021, Chad reported 833 animal infections (767 dogs and 66 cats), compared with 1,571 (1,508 dogs and 63 cats) reported in 2020 (Table 2). During January–June 2022, 43% fewer infected dogs (239) and 67% more infected cats (25) were reported compared with data from January–June 2021. In Chad, transmission of *D. medinensis* is hypothesized to have occurred from consumption of inadequately cooked fish, other aquatic transport hosts, or paratenic hosts (infected hosts in which the larval parasite does not develop) (*7*). As of June 2022, The Carter Center has been helping Chad’s Ministry of Health in implementing village-based surveillance for animal and human infections in 2,438 at-risk villages, an increase of 80 villages compared with the 2,358 in December 2021 (Table 1). The active surveillance generated 41,135 rumored reports about possible Guinea worm infections among humans or dogs during January–June 2022, a 33% decline compared with 61,341 rumored reports during the same period in 2021.

Since 2010, Chad’s Ministry of Health has offered a reward equivalent to US$100 for reporting a confirmed human dracunculiasis case. In addition, since 2013, Chad’s GWEP implemented educational interventions to avoid transmission through fish or fish entrails. Reporting and tethering dogs with dracunculiasis-compatible signs started in 2014, and this intervention was enhanced in 2015 with a US$20 equivalent reward for reporting confirmed infected dogs. In 2017, the program initiated the systematic use of temephos, focusing on small ponds in villages with the most infected dogs, and launched a nationwide communication campaign to increase awareness about the reporting and prevention of Guinea worm infection. In March 2020, Chad launched a new strategy to tether all dogs proactively during the 4 months of peak dracunculiasis incidence in all villages with five or more dracunculiasis infections during the previous year, and is currently working to expand proactive tethering among all villages reporting one or more dog infections.

Since June 2017, monitoring and evaluation efforts have indicated that approximately 81% of households assessed monthly in at-risk communities were burying fish entrails to avoid consumption by dogs, and 81% and 70% of eligible dogs were tethered in 2021 and during January–June 2022, respectively. The decrease in tethering coverage reflects the expansion of the intervention in 2022 to include all villages with one or more infections compared with 2021 when only villages with three or more infections were included. Water treatment with temephos reached 61% of 348 villages with dog (340) or human (eight) infections by December 2021 and 68% of 82 villages by June 2022. In December 2021, 79% of villages reporting infections among dogs or humans had at least one source of copepod-free drinking water. In areas under surveillance in Chad, 92% of residents surveyed in 2021, and 70% of those surveyed during January–June 2022 were aware of the cash rewards for reporting human or animal infections. These additional actions might be favorably affecting the elimination of dracunculiasis, and these effects will continue to be assessed.

**Cameroon.** Cameroon reported 10 infected dogs in 2021 and 23 during January–June 2022 (Table 2) in an area <3 miles (5 km) from the Chad-Cameroon border. Investigations indicate that dogs were likely infected in Chad, because the affected villages include families that permanently live on both sides of the border, and owners of infected dogs reportedly took their dogs to Chad during the period when the dogs would have become infected.

**Ethiopia.** During 2021, Ethiopia reported one human case and three infected animals (two dogs and one cat); no human case or infected animal was reported during January–June 2022 (Table 2), and for the first time in 9 years, no infected baboons were detected during January 2021–June 2022. For the past several years, all animal infections and human cases have occurred in Gog district of Gambella Region. Since 2017, The Carter Center has supported Ethiopia’s public health and wildlife authorities in a baboon and dog epidemiology project (*4*). Since 2021, the Ethiopia Dracunculiasis Eradication Program has conducted active surveillance of 198 villages and 201 nonvillage areas (e.g., commercial farms and temporary hunting settlements). The program applies temephos monthly to nearly all water sources known to have been used by humans in the at-risk area of Gog district. Beginning in 2022, remote sensing data from Maxar Technologies is identifying new water sources that need to be treated. Since April 2018, among villages in which infected animals were most commonly detected, Ethiopia has supported villager-initiated, constant tethering of approximately 1,900 dogs and cats to prevent their exposure to water sources in adjacent forests in which transmission apparently occurs (*4*). In 2018, Ethiopia increased its reward for reporting human dracunculiasis cases to an equivalent of US$360 and for reporting and tethering infected animals to US$40. In 2021, 92% of persons surveyed in active surveillance areas knew of the rewards; in January–May 2022, 96% were aware. In May 2022, the minister of health visited areas with endemic dracunculiasis to promote and support dracunculiasis eradication efforts.

**Mali.** Mali reported two human cases in 2021, and none during January–June 2022, compared with one case in 2020 and none during January–June 2021 (Table 2). In 2021, one infected cat and 16 infected dogs were reported, compared with nine dogs and no cats in 2020. During January–June 2022, Mali reported two confirmed infected dogs, the same number reported during January–June 2021. Among the 16 infected dogs identified in all of 2021, 10 were detected in Segou Region and six were detected in the adjacent Mopti Region. Segou Region is accessible to the program, but the dogs were bred and apparently became infected in areas of Mopti Region that have remained partly inaccessible to the program since 2012 because of civil unrest. The two infected dogs were detected and reportedly contained during January–June 2022 in Segou Region. In 2021 Mali had 2,216 villages under active surveillance (Table 1), with cash rewards equivalent to US$340 for reporting a human case and an equivalent of US$20 for reporting and tethering an infected animal. In areas under active surveillance in 2021, 90% of persons queried knew of the rewards for reporting an infected person or animal, and in January–June 2022, 92% and 81% knew of the rewards for reporting an infected person or animal, respectively. In addition, Mali introduced proactive tethering of some dogs late in 2021.

**South Sudan.** South Sudan reported four human cases in 2021, compared with one in 2020 (Table 2). No human cases or infected animals were reported during January–June 2021 or January–June 2022. Only one infected animal has ever been reported (in 2015), which belonged to a household in which a human infection had occurred. The high population mobility of cattle herders and others in South Sudan presents a special challenge, in addition to sporadic insecurity. By December 2021, South Sudan’s GWEP had 2,174 villages under active surveillance (Table 1). The reward for reporting a case of dracunculiasis or an infected animal is approximately equivalent to US$75. A 2021 survey of residents found 84% of respondents knew of the reward for reporting an infected person.

## Discussion

Stopping dracunculiasis transmission among dogs in Chad is now the principal challenge to the GWEP. During January 2021–June 2022, Chad reported 1,108 (94%) of the world’s 1,177 *D. medinensis* infections, 1,059 (96%) of which were in dogs. In a pattern peculiar to Chad, Guinea worm infections there usually affect many dogs and few humans (*7*). This challenge is being addressed through innovative interventions and research supported by The Carter Center, WHO, and CDC. Also involved are multiple research institutions helping to understand the unusual epidemiology of dracunculiasis in the remaining affected countries and assessing antihelminthic treatment of dogs (*8*). Research from the University of Georgia indicated that fish could serve as transport hosts for *Dracunculus* spp. and that *D. medinensis* can use frogs as paratenic hosts.^†††^ In addition, *Dracunculus* larvae have been recovered from multiple wild frogs in Chad (*9*,*10*). If the hypothesis that the parasite’s life cycle in Chad involves a transport or paratenic host is correct (*10*), increased active surveillance, proactive tethering of dogs, temephos application, and fish entrail burial should reduce transmission. The reductions in human cases and infected dogs in Chad in January 2021–June 2022 suggests that these interventions are successful.

Adequate security is important to achieving eradication goals, especially in Mali and South Sudan. In 2021, Mali reported two human cases, whereas the endemic transmission of Guinea worm among dogs and cats appears to be geographically limited. In 2020, the program in Chad began working with ministry of health, regional, and local leaders in a Peace-Health Initiative to reduce insecurity in one district with endemic dracunculiasis and expanded to four districts in 2022. South Sudan is poised to achieve zero-case status soon as a result of strong technical leadership, strong governmental political support, and no known animal infections, highlighting the importance of maintaining adequate security.

Areas of transmission in Ethiopia and Angola are limited. In 2021, Ethiopia reported one human case, and animal infections appear to be geographically limited. The ecologic study of baboons and proactive tethering of dogs in Gog district might help clarify the dynamics of residual Guinea worm infections in Ethiopia and identified the likely sources of all four infections in 2021. Now Ethiopia appears close to interrupting transmission. In Angola, three confirmed human cases and seven infected dogs reported between 2018 and 2022 suggest that the problem in this country is limited, but active surveillance throughout the areas at risk, and appropriate control measures and investigations are still needed.

The identification of dracunculiasis cases in villages in Cameroon that border areas in Chad with endemic dracunculiasis highlights the risks for exportation and the need for ongoing active surveillance and appropriate control measures in neighboring countries (Cameroon and Central African Republic). The current prominence of infections in domestic dogs and cats requires increased measures to limit those animals’ access to water sources for human consumption and waste from discarded fish and other aquatic animals.

With only 15 human cases identified in 2021 and three during January–June 2022, program efforts appear to be closer to reaching the goal of eradication. However, dog infections and impeded access because of civil unrest and insecurity in Mali and South Sudan continue to be the greatest challenges for the program.

**Summary**

**What is already known about this topic?**

Human cases of dracunculiasis (Guinea worm disease) have decreased from an estimated 3.5 million in 1986 to 15 in 2021. Emergence of Guinea worm infections in dogs in 2012 has complicated eradication efforts.

**What is added by this report?**

Fifteen cases in humans were reported in 2021 and three during January–June 2022. As of November 2022, dracunculiasis remained endemic in five countries (Angola, Chad, Ethiopia, Mali, and South Sudan).

**What are the implications for public health practice?**

With only 15 human cases identified in 2021 and three during January–June 2022, program efforts appear to be closer to reaching the goal of eradication. However, dog infections and impeded access because of civil unrest and insecurity in Mali and South Sudan continue to be the greatest challenges for the program.

## Footnotes

* Human cases are contained when all of the following criteria are met: 1) infected patients are identified ≤24 hours after worm emergence; 2) patients have not entered any water source since worm emergence; 3) a village volunteer/health care provider properly treats the lesion until all detectable worms are fully removed and educates the patient not to contaminate water sources; 4) the containment process is validated by a GWEP supervisor ≤7 days after worm emergence; and 5) all contaminated and potentially contaminated sources of drinking water are treated with temephos.

† A dracunculiasis case is defined as an infection occurring in a person exhibiting a skin lesion or lesions with emergence of one or more worms that is laboratory-confirmed as *Dracunculus medinensis* at CDC. Because *D. medinensis* has a 10–14-month incubation period, each infected person is counted as having a case only once during a calendar year.

§ Initially 20 countries, but the former country of Sudan officially separated into two countries (Sudan and South Sudan) on July 9, 2011.

¶
https://www.who.int/activities/eradicating-dracunculiasis

** Villages under active surveillance are those that have endemic dracunculiasis or are at high risk for importation. Active surveillance involves daily searches of households by village volunteers (supported by their supervisors) for persons or animals with signs of dracunculiasis.

†† An indigenous dracunculiasis human case or animal infection is defined as an infection consisting of a skin lesion or lesions with emergence of one or more Guinea worms in a person or animal who had no history of travel outside their residential locality during the preceding year.

§§
https://apps.who.int/iris/bitstream/handle/10665/354570/WER9721-22-eng-fre.pdf?sequence=1&isAllowed=y

¶¶ Specimens are laboratory-confirmed as *D. medinensis* at CDC by morphologic examination under a microscope or polymerase chain reaction assay. https://www.cdc.gov/dpdx/dxassistance.html

*** 45 C.F.R. part 46.102(l)(2), 21 C.F.R. part 56; 42 U.S.C. Sect. 241(d); 5 U.S.C. Sect. 552a; 44 U.S.C. Sect. 3501 et seq.

††† An intermediate host in which no parasite development occurs but which serves to maintain the viability of larval stages of a parasite.
